# “What’s in a Name?”

**Published:** 1888-03

**Authors:** 


### "WHAT'S IN A NAME?"

Some names become offensive or obtain great unpopularity with individuals and classes, on account of something obnoxious or distasteful associated with them. The primary signification may have been ever so innocent, even enticing, but if in the course of events it be made to stand for something quite unpopular, as an order or class, whose methods or doctrines are opposed to widely accepted views, it naturally, almost necessarily, shares the odium whether just or unjust, of that of which, by common usage, it has come to be technically descriptive, although equally so of many other things. This is particularly the case with the word "spiritualism." There is no religious society that would not resist the imputation of being *un*spiritual, and if not unspiritual, it follows, of course, that it must be spiritual, as, in its methods, hopes and aspirations elevated above the merely temporal. But now that we have large numbers of both sexes united in a system of belief altogether blameless and without offense, which asserts, nay, and in its practice demonstrates the actual communion of spirits with mortals in our day, as well as in the days of the past, to whom and to whose organized bodies the terms "spiritualist" and "spiritualism" are applied, these hitherto graceful words are avoided and in a sense outlawed by the simon-pure goody goodys, as descriptive of something quite inconsistent with the manner of their lives and professions. Thus, a little volume has been recently brought out by Ticknor & Co., of Boston, entitled "Light on the Hidden Way" with an introduction by the Rev. James Freeman Clarke, of less than a hundred words, wherein, he asserts, as if it were a matter for congratulation, that the author "had had no connection with so-called 'spiritualism,' and is unacquainted with any of the professional mediums," but he neglects to state that she is herself, not only a spiritualist, but a medium, and that her book is, in fact, a very clear and concise exposition of the spiritual philosophy and religion. Thus Mr. Clarke, from his clerical standpoint, repudiates the only name which is descriptive of its teachings. We did not require the assurance which Mr. Clarke makes that the author "is a sincere, truthful and conscientious woman." These qualities are apparent in her work. The first page of the prefatory chapter contains the following trueism: "No more strangers and exiles, but fellow citizens with the saints of the household of God, for Thou hast made one family, there and here, one living communion of seen and unseen."

The author has demonstrated that she is a sensitive with rare spiritual faculties of seeing and hearing—in spiritual parlance—a delicately organized clairvoyant and clairaudient medium. On a doctrinal point "It seems strange to me," she says "that those who profess to believe in immortal life, and to treasure the Bible, where angels ascending and descending hold familiar converse with men, and who, though believing in the transfiguration and resurrection of Jesus, are so averse to the idea of a continual communication between the two worlds, receive with coldness and unfaith the assurance that the friend called dead stands beside them most keenly alive. Thus, I have learned silence. But for how long? I wait the growth of this wonder seed. Something whispers to me that it shall bear precious fruit, from which may be distilled drops of healing for the sin sick and sorrowing."

And again, "That spiritualism in its purest and highest sense, is God's new dispensation to mankind, I do believe; and though tares are springing up with the wheat, a goodly harvest will, in time, be realized for the spiritual needs of the world."

The spirit communications presented in this little volume, as having been audibly given to its medium author, are obviously from a pure and highly intellectual source. The book, as a whole, is a compendium of the spiritual philosophy and religion pure and simple, and deserves to take its place as a text book of the "new dispensation."

Again, let us refer to the author's statements concerning her mediumship and methods. "While busy in my room, to-night, there came to me a venerable man, (spirit) a beautiful presence. He greeted me and said:" "You are rarely gifted, you hold a solemn trust, a light that should glorify your life." * * * Communicating further the celestial visitor went on to say that "There are some poor souls who go through life without learning a conscious lesson. Inherited tendencies, a lack of moral training, cruel circumstances and all manner of chilling influences would seem to have utterly blasted their spiritual natures. Yet the germ of good is there, dormant in its dull husk and here (in the spiritual world) shall be quickened into life. You know there are some seeds that will not germinate in the cold, open ground of northern latitudes, and that it is in a more genial atmosphere that they can be made to unfold. Sometimes it happens so with this germ soul; and here, in the divine nursery, not a seed is lost, but all wake to new possibilities.

The first thrill of life may be a terrible agony of remorse, the painful bursting of the hull. Then, for the first time, perhaps, comes to it a consciousness of what it is and what it might have been. A reaction from the belief in a literal hell has given many the very comfortable idea, that no matter how selfishly and unworthily they may have lived, at death their sins will be blotted out, that *then* they will begin to live better lives and enter into joy and peace. Nothing could be further from the truth."

On another occasion the same communicant said to her: "If we remember the number of undeveloped, evil minded and malicious souls passing constantly from our midst into the beyond, what more natural field presents itself for the exercise of their wicked propensities than the injury of those here on a higher plane than themselves, and towards whom they seem to feel a peculiar hatred and jealousy? We may be sure that the exact condition of our inner life and spiritual atmosphere is as clear to their vision as are physical forms to our mental senses, and that tendencies and moods are open avenues for the influence of these subtle workers to crowd in upon, and by their artful management of vacillating motives oftentimes to turn the wavering balance on the side of wrong and evil. How necessary, then, to keep the mind clear and the heart pure, that good angels may come in and help us to battle out every struggle for right. Presences, good and evil, are ever watching near us ; it is for ourselves to determine which shall be the chosen guests of our inner sanctuary."

Still again ; "The passive resignation which accepts everything as the will of God, is no longer possible to me ; the question continually arises as to how much misery is God's will, and how much the consequence of our ignorance and blindness. For instance, when loved members of a family die from the neglect of the common sanitary measures, does not the destroyer come into our homes as the penalty of broken laws, whether broken through wilful blindness or ignorance ? and is it not irreverent to say it is God's will that we suffer ? When the ship or railroad train, freighted with precious lives, is swept away through the carelessness of officials, or, it may be the incompetence of just one man, is it providential ? or are not these things allowed rather because God's laws are immutable, and we only learn to adjust ourselves to them by these solemn lessons ?" * * * *

"One who has my warmest sympathy was here a clergyman, a man of brilliant mind and natural liberal tendencies ; but rejecting the higher light, preached dogmas he no longer believed, closing his eyes and understanding to truths inconvenient to accept. His suffering is twofold, —the injury to his own spirit and a grief yet more intolerable over those misled by his teaching ; for he had many enthusiastic admirers, to whom his word and opinions were law."

I have found a most impressive fact to be that perfect *order* of the universe which causes every soul to find its own level and exact place, by the same unfailing law that builds the marvelous architecture of snow crystal and flower, and provides for the progress of each soul as surely as for the sweep of the planets in their mighty course. We are just beginning to read in the lessons of nature and history that providential order which we call "law."

There is great need of just such aids to truth as this admirable work presents, and it is destined to do great good in turning aside the thoughtless prejudice which prevents a candid examination into the truths of the "New Dispensation," on the part of those who are too firmly rooted in the superstitions of old theological systems, to give a patient hearing to anything that seems to controvert them, even though the former be utterly powerless against the rampant materialism of the present day.

"Life, like a dome of many colored glass,
Stains the white radiance of eternity."

